# Distinct roles of ATM and ATR in the regulation of ARP8 phosphorylation to prevent chromosome translocations

**DOI:** 10.7554/eLife.32222

**Published:** 2018-05-08

**Authors:** Jiying Sun, Lin Shi, Aiko Kinomura, Atsuhiko Fukuto, Yasunori Horikoshi, Yukako Oma, Masahiko Harata, Masae Ikura, Tsuyoshi Ikura, Roland Kanaar, Satoshi Tashiro

**Affiliations:** 1 Department of Cellular Biology, Research Institute for Radiation Biology and MedicineHiroshima UniversityHiroshimaJapan; 2 Department of Ophthalmology and Visual Science, Graduate School of Biomedical SciencesHiroshima UniversityHiroshimaJapan; 3 Laboratory of Molecular Biology, Graduate School of Agricultural ScienceTohoku UniversitySendaiJapan; 4 Laboratory of Chromatin Regulatory Network, Department of MutagenesisRadiation Biology Center, Kyoto UniversityKyotoJapan; 5 Department of Molecular GeneticsOncode InstituteRotterdamNetherlands; Stowers Institute for Medical ResearchUnited States

**Keywords:** ATM, ARP8 phosphorylation, chromosome translocations, INO80, RAD51, ATR, Human

## Abstract

Chromosomal translocations are hallmarks of various types of cancers and leukemias. However, the molecular mechanisms of chromosome translocations remain largely unknown. The ataxia-telangiectasia mutated (ATM) protein, a DNA damage signaling regulator, facilitates DNA repair to prevent chromosome abnormalities. Previously, we showed that ATM deficiency led to the 11q23 chromosome translocation, the most frequent chromosome abnormalities in secondary leukemia. Here, we show that ARP8, a subunit of the INO80 chromatin remodeling complex, is phosphorylated after etoposide treatment. The etoposide-induced phosphorylation of ARP8 is regulated by ATM and ATR, and attenuates its interaction with INO80. The ATM-regulated phosphorylation of ARP8 reduces the excessive loading of INO80 and RAD51 onto the breakpoint cluster region. These findings suggest that the phosphorylation of ARP8, regulated by ATM, plays an important role in maintaining the fidelity of DNA repair to prevent the etoposide-induced 11q23 abnormalities.

## Introduction

Chromosome translocations are one of the most common types of genetic rearrangements induced by DNA damaging agents, such as ionizing radiation and certain chemotherapies. The presence of disease-specific chromosome translocations, especially in hematological malignancies such as the t(9;22) or Philadelphia chromosome in chronic myelocytic leukemia, has been reported. Molecular studies of the breakpoints of such disease-specific chromosome translocations have revealed the clustering of the breakpoints in specific regions, designated as the breakpoint cluster region (BCR). In lymphoid malignancies, the involvement of the physiological recombination of immunoglobulin and T-cell receptor genes in chromosome translocations has been suggested, due to the presence of signal sequences for the recombination at the breakpoints. However, the molecular mechanisms of chromosome translocations in other cell types remain largely unknown.

Chromosome translocations arise as a consequence of errors in the repair of DNA double strand breaks (DSBs). Eukaryotic cells utilize a variety of repair pathways for DSBs, including two major ones, non-homologous end joining (NHEJ) and homologous recombination repair (HR). In the absence of these canonical pathways, the activation of the alternative NHEJ (Alt-EJ) pathway and the inactivation of DNA polymerase theta are implicated in chromosomal translocations (Zelensky et al., 2017) (Mizuno et al., 2009; Ruiz et al., 2009; Schmidt et al., 2006). In contrast, HR is regarded as a precise DSB repair system, since either the intact sister chromatid or the homologous region is used as the template for repair. However, both the depletion and overexpression of the RAD51 recombinase, a key factor involved in HR, lead to chromosomal abnormalities (Reliene et al., 2007; Richardson et al., 2004). Therefore, the precise regulation of the recombination activity is also required for DNA repair to prevent chromosome translocations.

DNA damage leads to the activation of the DNA damage response and repair pathways. The ataxia-telangiectasia mutated (ATM) protein regulates the DNA damage response in reaction to DSBs, through its kinase activity (Clouaire et al., 2017; Guleria and Chandna, 2016; Shiloh, 2003; Shiloh and Ziv, 2013). Alterations in the function of ATM play pathologic roles in the development of leukemia/lymphoma and cancer (Khanna, 2000; Oguchi et al., 2003; Reliene et al., 2007). Chromosome translocations involving the MLL gene on 11q23 are the most frequent chromosome abnormalities in secondary leukemia associated with chemotherapy employing etoposide, a topoisomerase II poison. An increase of 11q23 translocations is observed in the ATM kinase activity-deficient fibroblast cell line AT5BIVA (Nakada et al., 2006). We showed previously that a deficiency of ATM, a DNA damage signaling kinase, led to the excessive binding of RAD51 and the chromatin remodeling factor INO80 to the BCR in the MLL gene after etoposide treatment (Sun et al., 2010). INO80 is conserved in eukaryotes and acts as an integral scaffold for assembling other proteins into the INO80 chromatin remodeling complex (Chen et al., 2011; Morrison et al., 2004). The INO80 complex plays an important role in chromatin reorganization for transcription (Lafon et al., 2015; Xue et al., 2015), replication (Falbo and Shen, 2012; Vassileva et al., 2014) and DNA repair (Alatwi and Downs, 2015; Gospodinov et al., 2011; Morrison et al., 2004; Seeber et al., 2013; van Attikum et al., 2004). The INO80 complex is required for effective DNA end resection at the early stage of HR in budding yeast and human cells (Gospodinov et al., 2011; Lademann et al., 2017; Tsukuda et al., 2005; Tsukuda et al., 2009; van Attikum et al., 2004). Therefore, we speculated that the decreased fidelity of DNA repair by the inappropriate regulation of HR repair in ATM-deficient cells could lead to chromosomal translocations. However, the mechanism by which ATM deficiencies induce the excessive binding of INO80 and RAD51 to the BCR after etoposide treatment remains to be clarified.

INO80 forms a chromatin remodeling complex with more than 15 subunits (Jin et al., 2005; Shen et al., 2000). Among the subunits of the INO80 complex, ARP8 functions as a nucleosome recognition module and enhances the nucleosome-binding affinity of the protein complex (Saravanan et al., 2012; Shen et al., 2003). In this study, we found that ARP8 is required for the binding of INO80 and RAD51 to the BCR in the MLL gene after etoposide treatment. We also showed that the DNA damage-dependent phosphorylation of ARP8 is regulated by ATM and ATR after etoposide treatment. The ATM-dependent phosphorylation of ARP8 negatively regulates the interaction between INO80 and ARP8, leading to the reduced binding of INO80 and RAD51 to the BCR after etoposide treatment. In contrast, ATR was not involved in the regulation of the etoposide-induced binding of RAD51 to the BCR. These findings suggest that ATM plays distinct roles from ATR in the phosphorylation of ARP8 to prevent the etoposide-induced 11q23 chromosome translocations, through the negative regulation of INO80 and RAD51 binding to the BCR.

## Results

### Phosphorylation of ARP8 after etoposide treatment regulated by ATM and ATR

In our previous study, we showed that ATM deficiency resulted in the overloading of INO80 and RAD51 onto the BCR of the MLL gene after etoposide treatment (Sun et al., 2010). The finding led us to investigate the phosphorylation target of the ATM kinase, which could regulate the loading of INO80 and RAD51 onto the BCR. First, we examined the phosphorylation status of INO80 after etoposide treatment. However, we could not detect the phosphorylation of INO80 by ATM after etoposide treatment by an immunoblotting analysis (Figure 1—figure supplement 1A). Therefore, we decided to examine the phosphorylation of the subunits of the INO80 protein complex after etoposide treatment.

**Figure 1.**
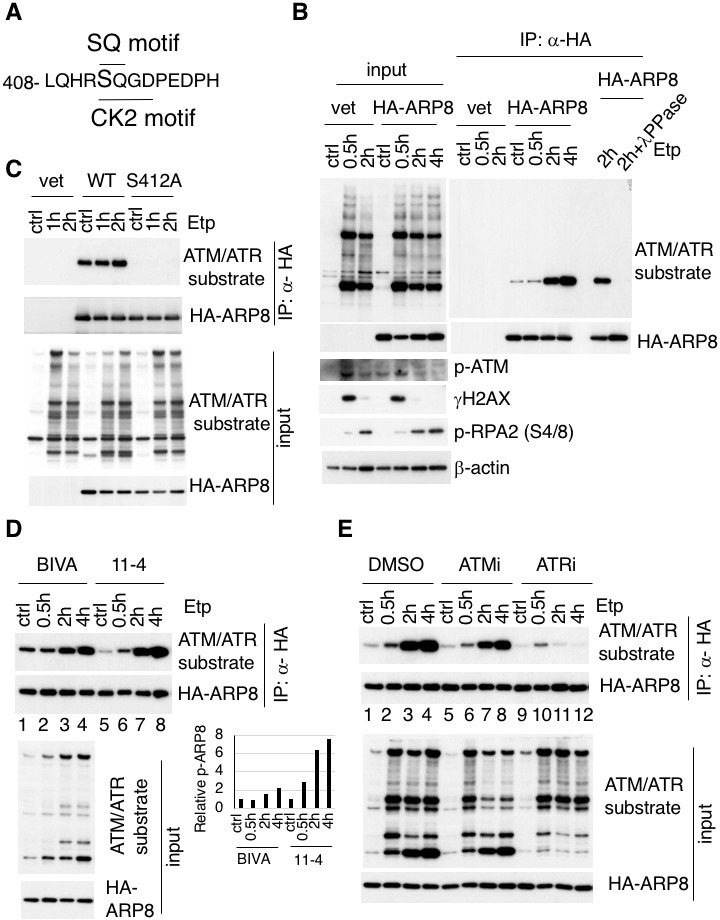
Identification of etoposide-induced ARP8 phosphorylation and the possible responsible kinase. (**A**) Amino acid sequence 408 through 420 of ARP8. The Ser412 residue, within the ATM/ATR substrate motif and the CK2 substrate motif, is indicated. (**B**) Immunoprecipitation analysis of ARP8 phosphorylation. U2OS cells transiently expressing an empty HA vector or a vector encoding HA-tagged ARP8 were treated with DMSO (ctrl) or etoposide (Etp) for 15 min, then washed twice and cultured in complete medium for the indicated times. The nuclear extracts were incubated with anti-HA-conjugated anti-mouse IgG Dynabeads. The precipitates were electrophoresed through a gel and probed by western blotting with an anti-ATM/ATR substrate antibody or an anti-HA antibody. λPPase treatment identified the band of phosphorylated HA-ARP8. The blot of input was probed by antibodies against phospho-ATM (p-ATM), γ H2AX or phospho-RPA2 at Ser4/8 (p-RPA2). β-actin was used as a loading control. (**C**) Identification of the ARP8 phosphorylation site by an immunoprecipitation analysis. U2OS cells were transfected with an empty HA vector (vet), or a vector encoding HA-tagged wild-type ARP8 (WT) or HA-ARP8 S412A (S412A) for 48 hr. The cells were washed after treatment with etoposide or DMSO for 15 min, cultured in fresh medium, and harvested at the indicated time points. Whole cell extracts were used for the immunoprecipitation analysis. (**D**) Etoposide-induced ARP8 phosphorylation in ATM-deficient BIVA and ATM-proficient 11–4 cells. Immunoprecipitation analysis of cell extracts of BIVA or 11–4 cells transfected with HA-tagged wild-type ARP8 using anti-HA antibodies. The cells were treated with DMSO (ctrl) or etoposide (Etp) for 15 min, cultured in fresh medium, and harvested at the indicated time points. Whole cell extracts were used for the immunoprecipitation analysis, which was performed as described in (**B**). The amounts of phosphorylated ARP8 and HA-ARP8 were quantified, using the Image J software. The results of the quantitative analysis are shown as the relative values to the DMSO controls. Source data are presented in Figure 1—source data 1. (**E**) Immunoprecipitation analysis of cell extracts from 11 to 4 cells expressing HA-tagged ARP8. The cells were treated with DMSO, 10 μM ATMi (KU55933), or 10 μM ATRi (VE821) for 2 hr before etoposide treatment, and then the inhibitors (5 μM) were added after the cells were washed. 10.7554/eLife.32222.005Figure 1—source data 1.Source raw data for Figure 1D.

The substrates of ATM contain the core sequence with an SQ or TQ motif (Kim et al., 1999; Matsuoka et al., 2007; O'Neill et al., 2000). By searching for SQ or TQ motifs in the subunits of the INO80 complex, we found that APR8 had an SQ motif at S412 and Q413. This motif in ARP8 is indicated as a putative phosphorylation site in the PhosphoSitePlus database (Figure 1A and Figure 1—figure supplement 1B–D). ARP8 is required for DNA binding by INO80 in yeast and mammals, and the ARP8 knockout in human cells impairs the binding of INO80 to chromatin and causes defects in DNA repair (Kashiwaba et al., 2010; Saravanan et al., 2012). Therefore, we examined whether ARP8 was the phosphorylation target of ATM. The immunoblotting analysis, using antibodies against the ATM/ATR substrate, revealed that the level of ARP8 phosphorylation was significantly increased from 2 hr after etoposide treatment (Figure 1B). The disappearance of the signal by a protein phosphatase treatment validated that the derived signal resulted from the phosphorylation of ARP8 (Figure 1B).

**Figure 1—figure supplement 1.**
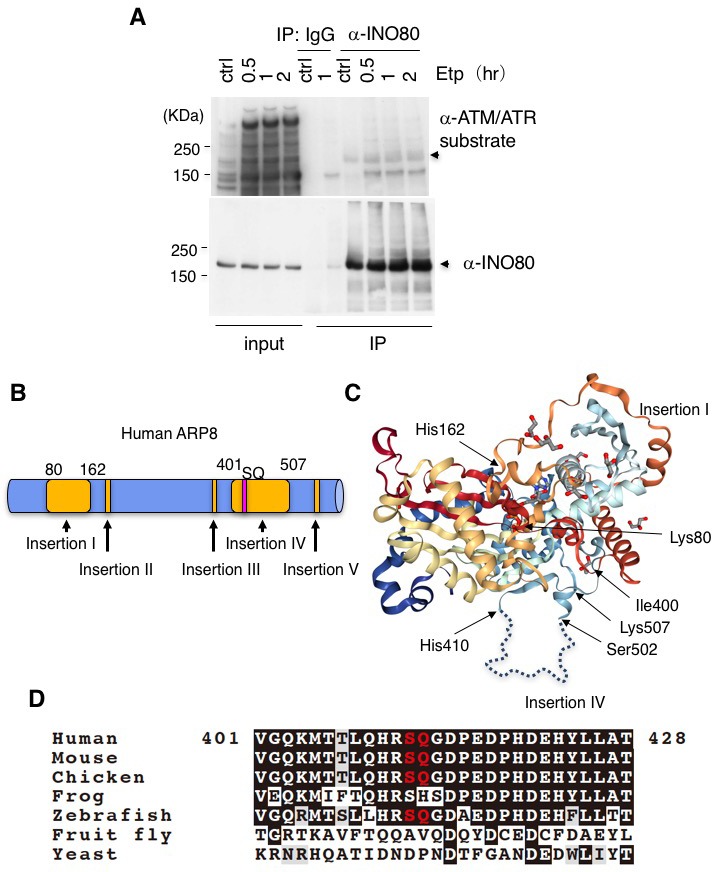
ARP8 contains an ATM/ATR substrate SQ motif at Ser412 and Q413. (**A**) Damage-induced INO80 phosphorylation was not detected by an ATM/ATR antibody. Immunoprecipitation analysis of INO80 phosphorylation. After treatment with or without etoposide for 15 min, cells were cultured in fresh medium for 0.5, 1 or 2 hr. The whole cell l extracts from U2OS cells were incubated with anti-INO80 or normal IgG-conjugated Dynabeads. The precipitates were electrophoresed through a gel and probed by western blotting with an anti-ATM/ATR substrate antibody or an anti-INO80 antibody. The arrows indicate the position of INO80. (**B**) Schematic diagram of full-length human ARP8. The insertions of ARP8 are shown in yellow, and the SQ motif in insertion IV is pink. (**C**) A 3D structure model of ARP8. Insertion I, insertion IV, and the locations of important amino acid residues are indicated. This structure was obtained from the Protein Database (PDB ID: 4FO0), and was modified according to the report by Gerhold et al. and the description for PDB ID: 4FO0. (**D**) Alignment of multiple ARP8 sequences. The partial amino acid sequences containing the SQ motif or the corresponding amino acids, which are colored red. The homologous sequence corresponding to human ARP8 was obtained from the NCBI Protein Database.

**Figure 1—figure supplement 2.**
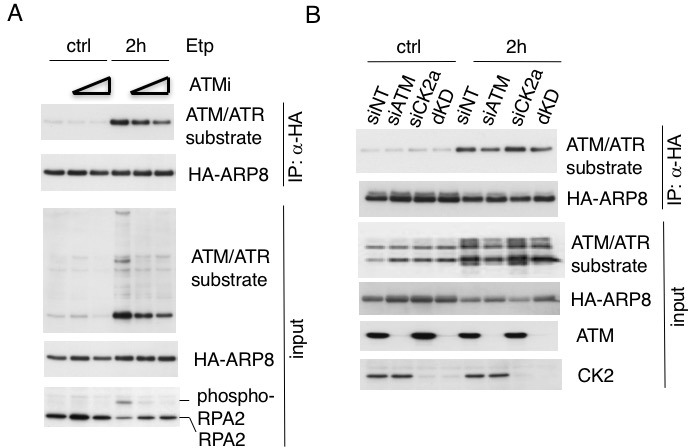
ATM, but not CK2 is responsible for ARP8 phosphorylation after etoposide treatment. (**A**) Immunoprecipitation analysis of U2OS cells transiently expressing HA-tagged ARP8, treated with or without an ATM inhibitor (ATMi). ATM inhibitor (10 or 20 μM) was added as indicated for 2 hr before etoposide (Etp) treatment. (**B**) Immunoprecipitation analysis of ARP8 phosphorylation, using U2OS cells cotransfected with HA-tagged ARP8 and either siATM, siCK2, or the double siRNAs (dKD) for 48 hr. The cells were cultured for 2 hr in fresh medium after etoposide (Etp) treatment. Immunoprecipitation was performed with anti-HA-conjugated beads. The precipitated proteins were detected by western blotting, using the indicated antibodies.

To confirm that Ser412 is the site in ARP8 that is phosphorylated after etoposide treatment, we produced an ARP8 mutant by replacing Ser412 with alanine. An immunoprecipitation analysis revealed that the etoposide-induced phosphorylation was completely abolished by the S412A substitution, indicating that Ser412 is the sole site within ARP8 that is phosphorylated in response to etoposide treatment (Figure 1C).

Next to investigate whether the etoposide-induced phosphorylation of ARP8 is regulated by ATM, we compared the phosphorylation status of ARP8 in ATM-deficient BIVA and ATM-proficient 11–4 cells after etoposide treatment. An immunoblotting analysis revealed that the etoposide-induced ARP8 phosphorylation in BIVA cells was lower than that in 11–4 cells (Figure 1D). Moreover, the phosphorylation of ARP8 in ATM-proficient 11–4 cells after etoposide treatment was repressed by the ATM-specific inhibitor, KU55933 (ATMi) (Figure 1E; lanes 1–8). These findings suggest that ATM regulates the ARP8 phosphorylation after etoposide treatment. The dose-dependent repression of the etoposide-induced ARP8 phosphorylation by ATMi in U2OS cells further supported this notion (Figure 1—figure supplement 2A).

Since ATMi did not completely abolish the etoposide-induced phosphorylation of ARP8, we next examined the relevance of ATR, another PI3-family kinase member sharing the same phosphorylation motif with ATM. ATR is responsible for the phosphorylation in the DNA replication stress response, and is activated by ATM after ionizing radiation (Cuadrado et al., 2006; Jazayeri et al., 2006; Myers and Cortez, 2006). The etoposide-induced phosphorylation of ARP8 was strongly repressed by the ATR inhibitor VE821 (ATRi) in 11–4 cells (Figure 1E, lanes 9–12). This finding suggests that ATR is the major kinase responsible for etoposide-induced ARP8 phosphorylation. In contrast, casein kinase 2, another kinase involved in the DNA damage response (Olsen et al., 2012) (Guerra et al., 2014) was not involved in the phosphorylation of ARP8 after etoposide treatment (Figure 1—figure supplement 2B). Together, these findings suggest that the phosphorylation of ARP8 at S412 after etoposide treatment is regulated by ATM and ATR.

### Negative regulation of the etoposide-induced loading of INO80 onto the MLL BCR by the phosphorylation of ARP8

**Figure 2.**
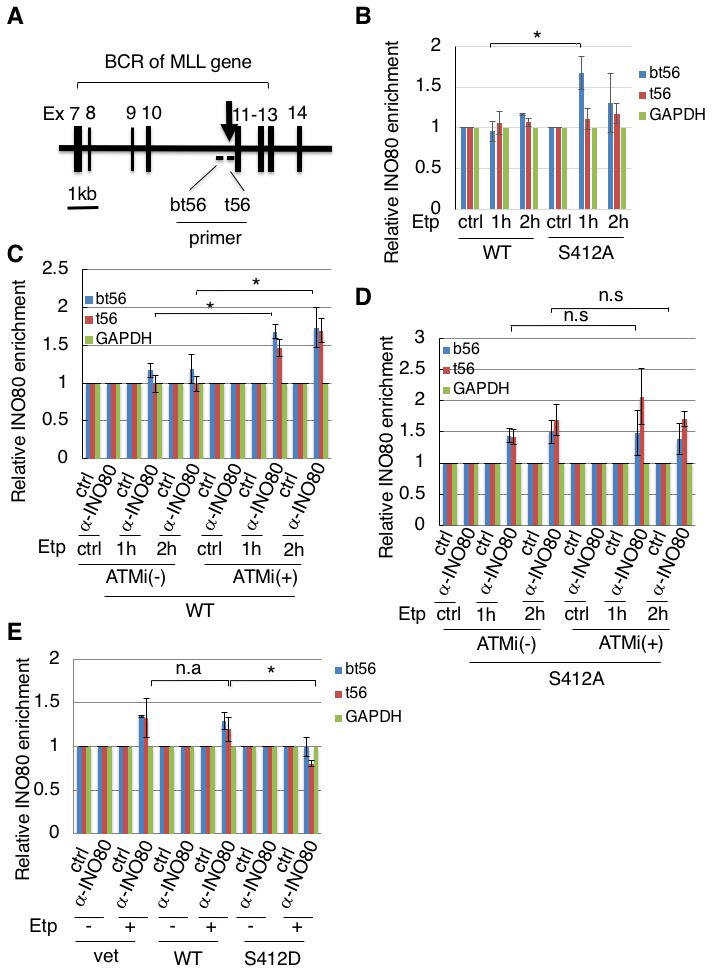
Phosphorylation of ARP8 negatively regulates the etoposide-induced enrichment of INO80. (**A**) Schematic representation of the BCR in the MLL gene. The locations of the primers used in the real-time PCR analyses are shown. The arrow indicates 11q23 chromosome translocation breakpoint hotspot identified in treatment-related leukemia. Ex: Exon. (**B**) ChIP analysis of the INO80 loading onto the MLL BCR in endogenous ARP8-depleted11-4 Flp-In cells expressing either the siRNA-resistant wild-type (WT) or phospho-deficient ARP8 (S412A) after tetracycline treatment. The cells were treated with DMSO (ctrl) or etoposide for 15 min, washed, and then cultured in fresh medium for 1 or 2 hr. GAPDH is shown as the control region. Values represent the means ± SE from three independent experiments. *: p<0.05. Source data are presented in Figure 2—source data 1. (**C**) ChIP analysis of the INO80 loading onto the MLL BCR in wild-type ARP8 expressing11-4 Flp-In cells. The cells were treated with/without an ATM inhibitor (KU55933) for 2 hr before etoposide treatment, and then the inhibitors (5 μM) were added after the cells were washed. The experiment was performed as described in (**B**). Values represent the means ± SE from three independent experiments. *: p<0.05. The level of ATM phosphorylation or expression of INO80 was shown in Figure 2—figure supplement 2B. Source data are presented in Figure 2—source data 1. (**D**) ChIP analysis of the INO80 loading onto the MLL BCR in S412A ARP8 expressing 11–4 Flp-In cells. The cells were treated with/without 10 μM ATM inhibitor for 2 hr before etoposide treatment, and then the inhibitors (5 μM) were added after the cells were washed. The experiment was performed as described in (**B**). Values represent the means ± SE from three independent experiments. n.s: no significant difference. The levels of ATM phosphorylation and INO80 expression are shown in Figure 2—figure supplement 2C. Source data are presented in Figure 2—source data 1. (**E**) ChIP analysis of the INO80 loading onto the MLL BCR in endogenous ARP8-depleted BIVA cells transfected with either the siRNA-resistant wild-type (WT) or phospho-mimetic ARP8(S412D). The control cells were transfected with an empty vector and a non-targeting siRNA (vet). The cells were treated with DMSO (ctrl) or etoposide for 15 min, washed, and then cultured in fresh medium for 1 hr. Values represent the means ± SE from three independent experiments. *p<0.05, n.a: not analyzed. Source data are presented in Figure 2—source data 1. 10.7554/eLife.32222.009Figure 2—source data 1.Source raw data for Figure 2B-E. 10.7554/eLife.32222.010Figure 2—source data 2.Source raw data for Figure 2—figure supplement 1A and B.

Having established that ARP8 is phosphorylated after etoposide treatment, we investigated the role of ARP8 in INO80 loading onto the MLL BCR (Figure 2A). The enrichment of γH2AX on BCR was observed in ATM-proficient 11–4 cells after etoposide treatment, suggesting that etoposide induces DNA damage specifically at the BCR (Figure 2—figure supplement 1A). A chromatin immunoprecipitation (ChIP) analysis revealed that the depletion of ARP8 reduced the binding of INO80 to the BCR after etoposide treatment in BIVA cells ((Figure 2—figure supplement 1B). Since the depletion of ARP8 did not affect the levels of INO80 (Figure 2—figure supplement 1C), the result suggests that ARP8 is required for loading INO80 onto the MLL BCR after etoposide treatment in BIVA cells. Next, to explore the role of ARP8 phosphorylation in the regulation of INO80 loading onto the BCR after etoposide treatment, we generated ATM-proficient 11–4 cell lines expressing the siRNA-resistant wild-type ARP8 (WT) or the phosphorylation-deficient mutant S412A (Figure 2—figure supplement 2A). The ChIP analysis revealed that the expression of the S412A mutant in ATM-proficient cells increased the binding of INO80 to the BCR after etoposide treatment (Figure 2B). Moreover, ATMi treatment increased the binding of INO80 to the BCR after etoposide treatment in ATM-proficient cells expressing wild-type ARP8 (Figure 2C). These findings suggest that the etoposide-induced phosphorylation of ARP8 represses the binding of INO80 to the BCR. Importantly, the ATMi treatment failed to enhance the enrichment of INO80 at the BCR in ATM-proficient 11–4 cells expressing the ARP8 S412A mutant (Figure 2D). This suggests that ARP8 phosphorylation at S412 regulated by ATM is responsible for the excessive binding of INO80 at the BCR after etoposide treatment. To further confirm the repression of the INO80 binding to the BCR by the ARP8 phosphorylation, we next introduced the siRNA-resistant phosphomimetic mutant S412D into the ATM-deficient BIVA cells. Consistently, the expression of the phosphomimetic mutant S412D in BIVA cells reduced the binding of INO80 to the BCR after etoposide treatment (Figure 2E). Taken together, these results indicate that the phosphorylation of ARP8 represses the loading of INO80 onto the MLL BCR in response to etoposide-induced damage.

**Figure 2—figure supplement 1.**
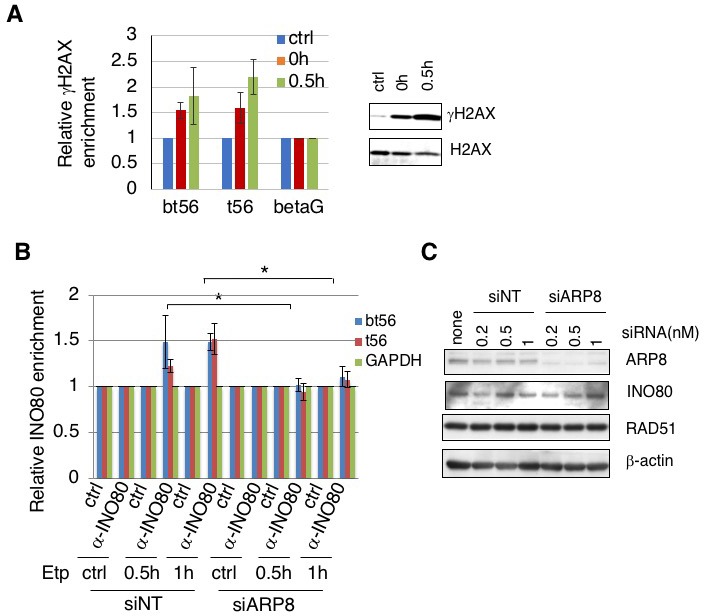
(**A**) Etoposide treatment induced H2AX phosphorylation on BCR of MLL gene. ChIP analysis of γH2AX enrichment on the MLL BCR in ATM proficient 11–4 cells. The cells were treated with DMSO (ctrl) or etoposide for 15 min, washed, and then fixed immediately (0 hr) or after culture in fresh medium for 0.5 hr (0.5 hr). β-globin (betaG) is shown as the control region. Values represent the means ± SE from four independent experiments. The results of immunoblotting analyses of γH2AX and H2AX are shown. The cells were treated with DMSO (ctrl) or etoposide for 15 min, washed, and then harvested immediately (0 hr) or after culture in fresh medium for 0 (Etp) or 0.5 hr (0.5 hr). Source data are presented in Figure 2—source data 2. (**B**) Depletion of ARP8 reduced the etoposide-induced enrichment of INO80 onto the BCR of the MLL gene. ChIP analysis of INO80 loading onto the MLL BCR in AT5BIVA cells depleted by either a non-targeting siRNA (siNT) or siARP8. The cells were treated with DMSO (ctrl) or etoposide for 15 min, washed, and then cultured in fresh medium for 30 or 60 min. GAPDH is shown as the control region. Values represent the means ± SE from four independent experiments. *: p<0.05. Source data are presented in Figure 2—source data 2. (**C**) ARP8 depletion did not affect INO80 or RAD51 expression. Immunoblotting analysis of non-targeting siRNA(NT) or siARP8 transfected cells. The final concentration of each siRNAs is indicated. β-actin was used as the loading control. In further studies, we used 0.2–0.3 nM of siRNA. The data showed that the depletion of ARP8 did not disturb INO80 and RAD51 levels.

**Figure 2—figure supplement 2.**
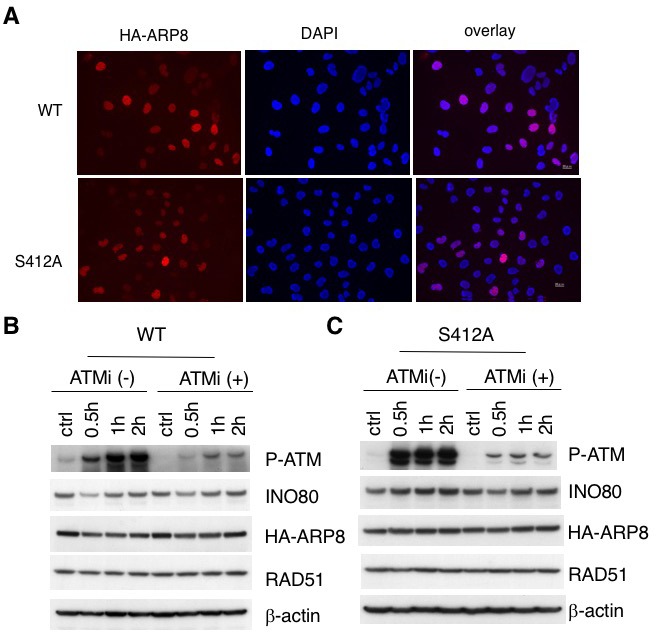
Establishment of stable and inducible ARP8 expressing 11–4 Flp-In cells. (**A**) Immunofluorescence staining of 11–4 Flp-In cells expressing HA-tagged wild-type ARP8 (WT) or S412 phospho-deficient ARP8 (SA). For induction of HA-ARP8 expression, tetracycline was added for 24 hr. The anti-HA antibody was used to identify the expression of HA-ARP8. DAPI was used for DNA staining. (**B and C**) Immunoblotting analysis of phosphorylated-ATM, and expression level of INO80 and RAD51 in 11–4 Flp-In cells. The endogenous ARP8-depleted11-4 Flp-In cells with tetracycline inducible expression of siRNA-resistant wild-type (WT) (**B**) or S412A ARP8 (S412A) (**C**), were treated with/without 10 μM ATM inhibitor for 2 hr, before DMSO (ctrl) or etoposide treatment. The cells were harvested at the indicated time point after etoposide removal. β-actin is shown as a loading control.

### Phosphorylation of ARP8 regulates its interaction with INO80

**Figure 3.**
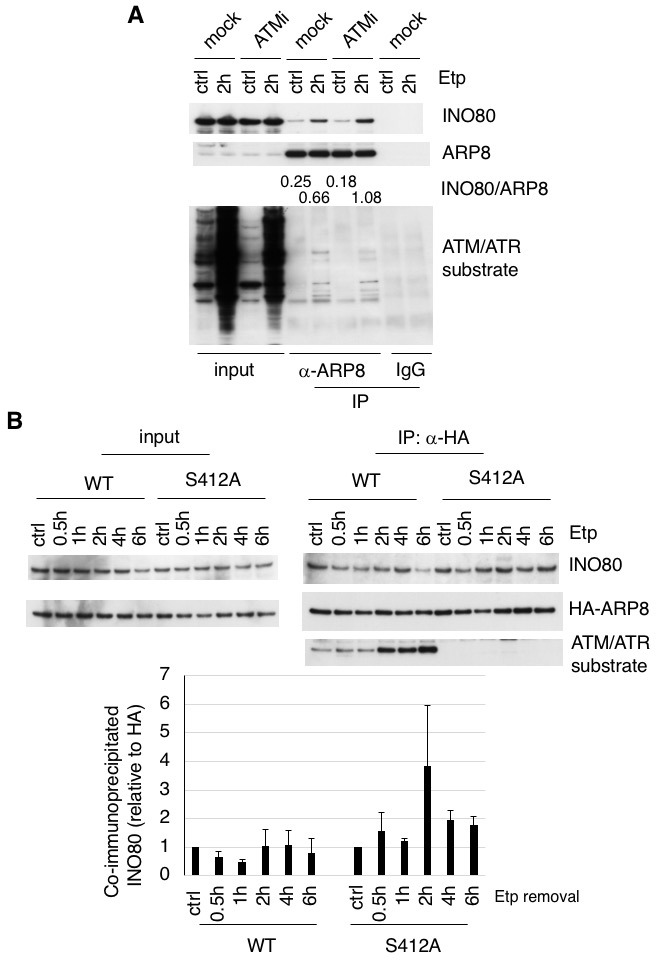
ARP8 phosphorylation deficiency increased its interaction with INO80. (**A**) Immunoprecipitation analysis of the interaction between INO80 and ARP8 in ATM inhibitor treated U2OS cells. The cells were treated with 10 μM KU55933 (ATMi) or equal amounts of DMSO (mock) for 2 hr, and then treated with DMSO (ctrl) or etoposide for 15 min, washed, and then cultured in fresh medium with or without 5 μM KU55933 for 2 hr. Immunoprecipitation analysis was performed with either anti-ARP8 antibodies or normal IgG. The relative immunoprecipitated amounts of INO80 are shown. Quantitative analysis was performed using the Image J software. (**B**) Examination of the interaction between INO80 and ARP8 in U2OS cells expressing HA-tagged wild-type or S412A ARP8. The endogenous ARP8-depleted cells were treated with etoposide for 15 min. After the cells were washed, they were placed in fresh medium and harvested at the indicated time points. The nuclear extracts were incubated with anti-HA-conjugated anti-mouse IgG Dynabeads. The precipitates were electrophoresed through a gel and probed by western blotting with an anti-INO80 or an anti-HA or an anti-ATM/ATR substrate antibody. The amounts of INO80 and HA-ARP8 were quantified, using the Image J software. The results of quantitative analysis are shown as the relative values as compared to the DMSO control from three independent experiments. Source data are presented in Figure 3—source data 1. 10.7554/eLife.32222.014Figure 3—source data 1.Source raw data for Figure 3B. 10.7554/eLife.32222.015Figure 3—source data 2.Source raw data for Figure 3—figure supplement 1B. 10.7554/eLife.32222.016Figure 3—source data 3.Source raw data for Figure 3—figure supplement 2B.

To determine whether the phosphorylation of ARP8 affects the loading of INO80 onto the MLL BCR through its interaction with INO80, we performed an immunoprecipitation analysis using U2OS cells. We found that etoposide treatment increased the interaction between INO80 and ARP8 in the ATM-proficient U2OS cells (Figure 3A). The etoposide-induced interaction between INO80 and ARP8 was enhanced by the treatment of U2OS cells with an ATM inhibitor (Figure 3A). These findings raised the possibility that ATM negatively regulates the interaction of ARP8 with INO80 after etoposide treatment. Therefore, we investigated the role of the ATM-regulated phosphorylation of ARP8 in the interaction between ARP8 and INO80 after etoposide treatment by using the U2OS cells stably expressing the phosphorylation-deficient ARP8 S412A mutant. An immunoprecipitation analysis showed an increased level of interaction of the ARP8 S412A mutant with INO80 after etoposide treatment, as compared to that of wild-type ARP8 (ARP8 WT) in ATM-proficient cells (Figure 3B). Similar results were obtained using the U2OS cells transiently expressing the ARP8 S412A mutant (Figure 3—figure supplement 1A). These findings support the notion that the phosphorylation of ARP8 represses its interaction with INO80 after etoposide treatment. To confirm the repression of the association of ARP8 with INO80 by ATM, we transiently expressed HA-tagged ARP8 WT or S412D mutant in ATM-deficient BIVA cells. An immunoprecipitation analysis revealed the increased interaction of INO80 with ARP8 WT, but not with the S412D mutant, in ATM-deficient BIVA cells after etoposide treatment (Figure 3—figure supplement 1B). A proximity ligation assay confirmed the decreased interaction of INO80 with the ARP8 S412D mutant (Figure 3—figure supplement 2). Taken together, these findings suggest that the phosphorylation of ARP8 represses the interaction between INO80 and ARP8 after etoposide treatment.

**Figure 3—figure supplement 1.**
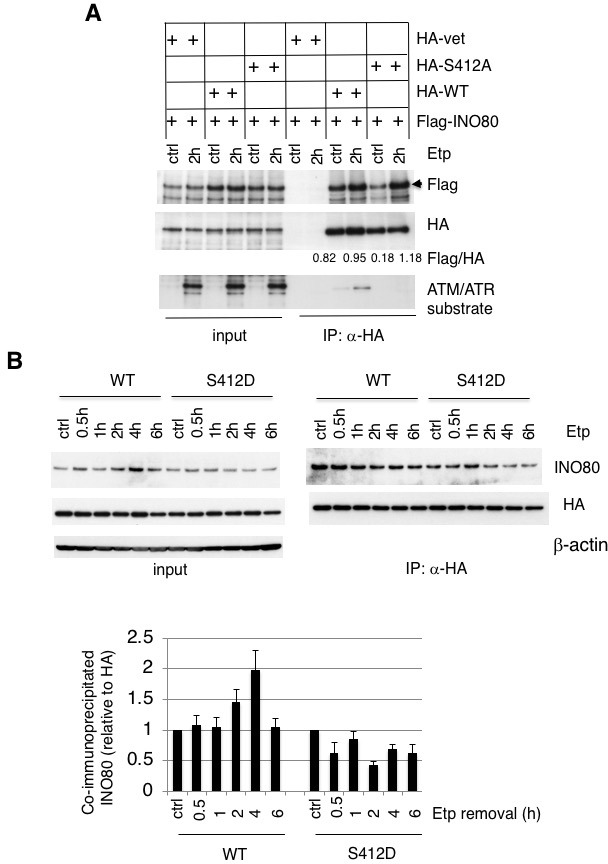
Examination of the interaction between ARP8 and INO80. (**A**) Examination of the interaction between INO80 and wild-type ARP8 or phospho-deficient ARP8. U2OS cells were co-transfected with Flag-INO80 and HA (vet), HA-ARP8 (WT), or HA-ARP8 S412A vectors, respectively, for 48 hr and subsequently treated with DMSO (ctrl) or etoposide for 15 min, washed, and then cultured in fresh medium for 2 hr. The immunoprecipitation analysis was performed using anti-HA antibodies. The precipitates were electrophoresed through a gel and probed by western blotting with an anti-Flag or an anti-HA or anti-ATM/ATR substrate antibody. The co-immunoprecipitated amounts of Flag-INO80 relative to HA-ARP8 are shown. Quantitative analysis was performed using the Image J software. (**B**) Co-immunoprecipitation analysis of ARP8 and INO80. ATM-deficient BIVA cells were co-transfected with the siARP8 and siARP8-resistant HA-tagged wild-type ARP8 or phospho-mimetic S412D ARP8 mutant. After etoposide removal, the cells were recovered at the indicated time points. The nuclear extracts were incubated with anti-HA-conjugated anti-mouse IgG Dynabeads. The precipitates were electrophoresed through a gel and probed by western blotting with either an anti-INO80 antibody or an anti-HA antibody. The amounts of INO80 and HA-ARP8 were quantified, using the Image J software. The results of quantitative analysis are shown as the relative values as compared to the DMSO control, from three independent experiments. Source data are presented in Figure 3—source data 2.

**Figure 3—figure supplement 2.**
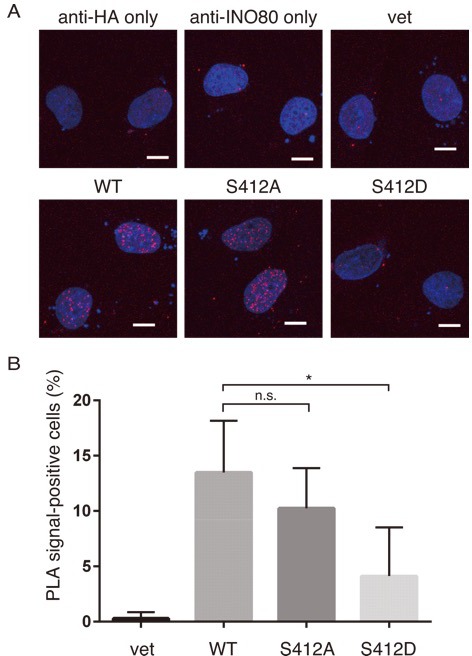
Examination of ARP8-INO80 interactions using Proximity ligation assay. (**A**) Proximity ligation assay (PLA) signals (in red) indicate ARP8-INO80 interactions. GM0637 cells were transfected with the HA-tagged wild-type, S412 A or S412D mutant of ARP8 or empty vector (vet). Nuclei were stained with DAPI (in blue). Scale bar: 10 μm. (**B**) Percentages of cells with more than five PLA signals. All results are the means of four independent experiments, and error bars show the standard deviation of the mean. *p<0.05. n.s.: no significant difference. Source data are presented in Figure 3—source data 3.

### ARP8 phosphorylation negatively regulates RAD51 loading onto the BCR after etoposide treatment

In our previous study, we detected the excess loading of RAD51 onto the BCR of the MLL gene in etoposide-treated ATM-deficient BIVA cells (Sun et al., 2010). In yeast, INO80 promotes DNA end resection and RAD51 binding to ssDNA for homology search/invasion during HR (Lademann et al., 2017; Tsukuda et al., 2009). To explore the mechanism of excessive RAD51 loading on the BCR, we examined the involvement of INO80 in RAD51 binding to damaged chromatin. A ChIP assay revealed that the depletion of INO80 by siRNA reduced the loading of RAD51 onto the MLL BCR in BIVA cells after etoposide treatment, suggesting that human INO80 promotes RAD51 binding to the BCR (Figure 4—figure supplement 1A and B). We next investigated the requirement for ARP8 in RAD51 binding to the MLL BCR. A ChIP analysis showed that the depletion of ARP8 reduced the binding of RAD51 to the MLL BCR in BIVA cells after etoposide treatment (Figure 4—figure supplement 1C). Since the depletion of ARP8 did not affect the level of RAD51 (Figure 2—figure supplement 1C), these findings suggest the involvement of ARP8 in the excessive binding of RAD51 to the BCR in BIVA cells.

**Figure 4.**
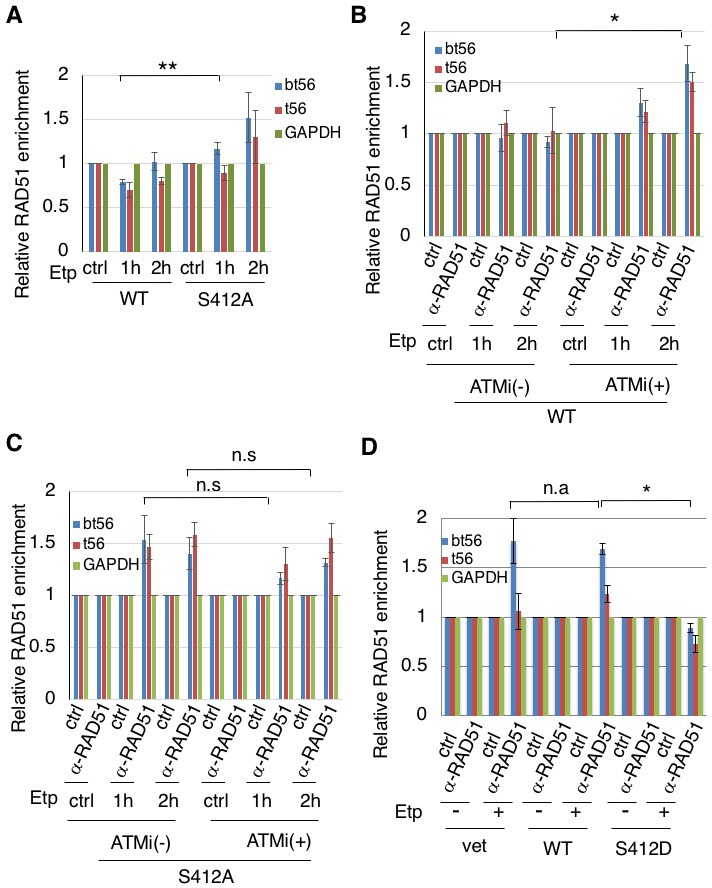
ARP8 phosphorylation prevents the excessive RAD51 loading onto MLL BCR. (**A**) ChIP analysis of the RAD51 loading onto the MLL BCR in endogenous ARP8-depleted11-4 Flp-In cells expressing the siRNA-resistant wild-type (WT) or phospho-deficient ARP8 (S412A) after tetracycline treatment. The cells were treated with DMSO (ctrl) or etoposide for 15 min, washed, and then cultured in fresh medium for 1 or 2 hr. Values represent the means ± SE from three independent experiments. **: p<0.01. Source data are presented in Figure 4—source data 1. (**B**) ChIP analysis of the RAD51 loading onto the MLL BCR in wild-type ARP8 expressing 11–4 cells. Following a treatment with/without 10 μM ATM inhibitor (KU55933) for 2 hr, the cells were treated with DMSO (ctrl) or etoposide for 15 min, washed, and then cultured in fresh medium with or without 5 μM KU55933 for 1 or 2 hr. Values represent the means ± SE from three independent experiments. *p<0.05. The levels of ATM phosphorylation and expression of RAD51 are shown in Figure 2—figure supplement 2B. Source data are presented in Figure 4—source data 1. (**C**) ChIP analysis of the RAD51 loading onto the MLL BCR in S412A ARP8 mutant expressing 11–4 Flp-In cells. The experiment was performed as described in (**B**). Values represent the means ± SE from three independent experiments. n.s: no significant difference. The levels of ATM phosphorylation and RAD51 expression are shown in Figure 2—figure supplement 2C. Source data are presented in Figure 4—source data 1. (**D**) ChIP analysis of the RAD51 loading onto the MLL BCR in endogenous ARP8-depleted BIVA cells transfected with either the siRNA-resistant wild-type (WT) or phospho-mimetic ARP8 (S412D). GAPDH is shown as the control region. The control cells were transfected with the empty vector and the non-targeting siRNA (vet). Values represent the means ± SE from three independent experiments. *p<0.05. n.a: not analyzed. Source data are presented in Figure 4—source data 1. 10.7554/eLife.32222.019Figure 4—source data 1.Source raw data for Figure 4A-D. 10.7554/eLife.32222.020Figure 4—source data 2.Source raw data for Figure 4—figure supplement 1B and C.

We then studied the effect of ARP8 phosphorylation on the regulation of RAD51 binding to the BCR after etoposide treatment. The expression of the ARP8 phosphorylation-deficient mutant S412A increased the RAD51 binding to the BCR of the MLL gene after etoposide treatment in ATM-proficient cells (Figure 4A). In ATM-proficient cells, ATMi treatment resulted in an increase of RAD51 binding to the BCR (Figure 4B), but it did not cause a further increase of RAD51 when ARP8 S412A was expressed (Figure 4C). In contrast, the expression of the ARP8 phosphomimetic mutant S412D in ATM-deficient BIVA cells repressed the excessive binding of RAD51 to the BCR (Figure 4D). These results strongly suggest that ARP8 phosphorylation at S412 represses the loading of RAD51 onto the MLL BCR after etoposide treatment. Taken together, the phosphorylation of ARP8 regulated by ATM may negatively regulate the loading of RAD51 onto the BCR after etoposide treatment, by repressing the loading of the INO80 complex.

**Figure 4—figure supplement 1.**
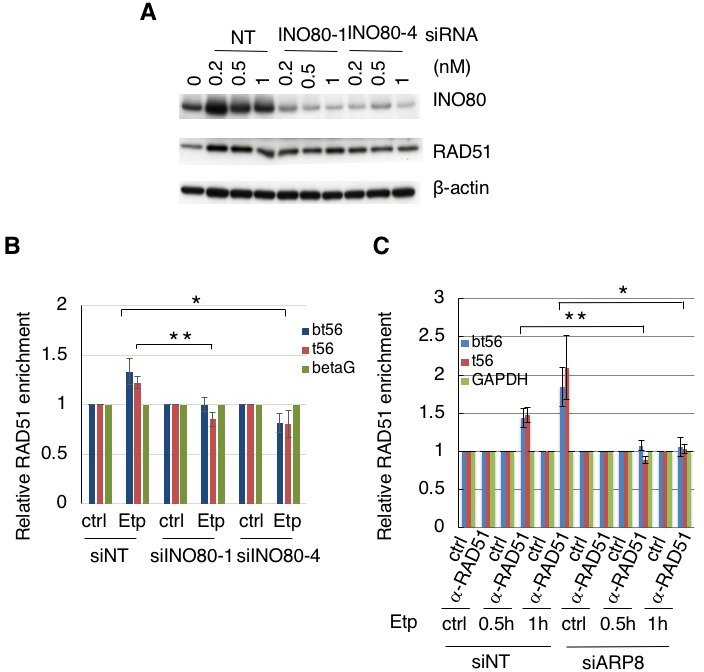
Requirement of INO80 and ARP8 for RAD51 binding to the BCR of the MLL gene. (**A**) Immunoblotting analysis of INO80 depletion efficiency. Two kinds of siRNAs against INO80 were used for the detection of the INO80 knockdown efficiency. RAD51 expression did not affect the INO80 knockdown. β−actin was used as the loading control. (**B**) ChIP analysis of the enrichment of RAD51 in AT5BIVA cells treated with non-targeting siRNA (siNT) or the siRNAs against INO80 (siINO80-1 and siINO80-4). The cells were treated with etoposide for 15 min, washed, and then cultured in fresh medium for 1 hr (Etp). Vehicle treatment was performed as a control. The DNA was analyzed by real-time PCR, using the MLL BCR gene primers. The β−globin gene (betaG) was used as a control region. Values represent the means ± SE from six independent experiments. *p<0.05. Source data are presented in Figure 4—source data 2. (**C**) ChIP analysis of RAD51 loading onto the MLL BCR, in AT5BIVA cells transfected with the non-targeting siRNA (siNT) or siARP8. The cells were treated with DMSO (ctrl) or etoposide for 15 min, washed, and then cultured in fresh medium for 30 or 60 min. GAPDH is shown as the control region. Values represent the means ± SE from four independent experiments. *p<0.05, **p<0.01. Source data are presented in Figure 4—source data 2.

### Repression of 11q23 chromosome translocations through the phosphorylation of ARP8

**Figure 5.**
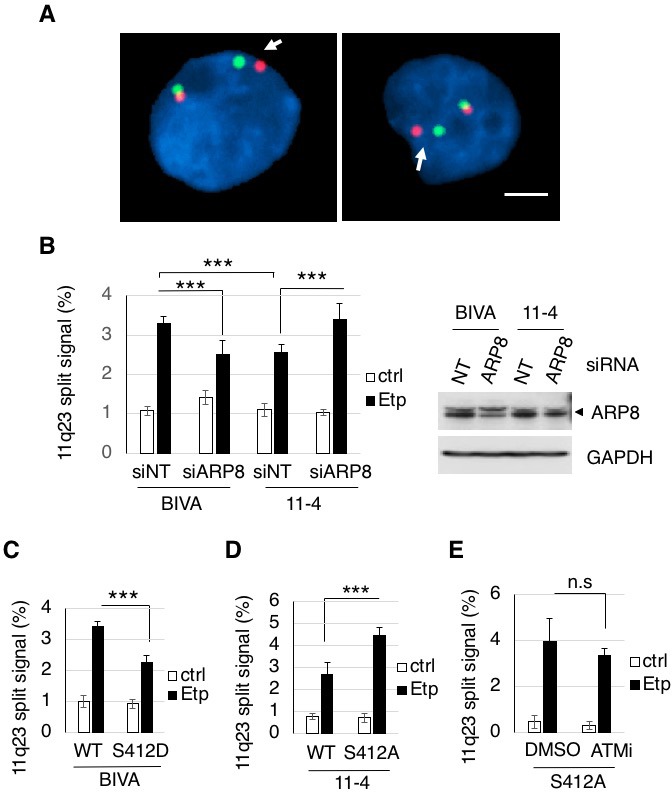
ARP8 phosphorylation averts 11q23 chromosome translocations. (**A**) Dual-color FISH analysis of chromosome 11q23. Representative FISH images using etoposide treated BIVA cells are shown. Arrows indicate the split signals (separated by >1 µm). Scale bar: 5 μm. (**B**) The percentages of AT5BIVA or 11–4 cells with split chromosome 11q23 signals are shown. The non-targeting control siRNA (siNT) or siARP8-depleted cells were treated with DMSO (ctrl) or etoposide for 15 min, washed, and cultured for 6 hr in fresh medium. At least 2,000 cells were analyzed in every experiment. The average percentages of cells with split signals from four independent experiments are shown. Values represent the means ± SE. ***p<0.001 as determined by the Z test. The ARP8 knockdown is shown in the gel image on the right. Source data are presented in Figure 5—source data 1. (**C, D**) Dual-color FISH analyses of chromosome 11q23 using ARP8-depleted AT5BIVA (**C**) and 11–4 (**D**) cells expressing the siARP8-resistant ARP8 wild-type (WT), S412D, or S412A. The average percentages of the cells with split signals from three independent experiments are shown. Values represent the means ± SE. ***p<0.001 as determined by the Z test. Source data are presented in Figure 5—source data 1. (**E**) Dual-color FISH analyses of chromosome 11q23 using 11–4 cells expressing the siARP8-resistant ARP8 S412A. The cells were treated with/without 10 μM ATM inhibitor (KU55933) for 2 hr before etoposide treatment. After the cells were washed, KU55933 (5 μM) was added until the cells were harvested. The average percentages of the cells with split signals from three independent experiments are shown. Values represent the means ± SE from three independent experiments. n.s.: no significant difference. Source data are presented in Figure 5—source data 1. 10.7554/eLife.32222.023Figure 5—source data 1.Source raw data for Figure 5B-E. 10.7554/eLife.32222.024Figure 5—source data 2.Source raw data for Figure 5—figure supplement 1A and B.

Having established that ARP8 regulates the loading of RAD51 and INO80 onto the BCR after etoposide treatment, we decided to examine the involvement of ARP8 in 11q23 chromosome translocations. A two-color fluorescence in situ hybridization (FISH) analysis, covering the upstream and downstream regions of the MLL BCR, revealed that the number of BIVA cells carrying split FISH signals after etoposide treatment was significantly reduced by the siRNA-mediated depletion of ARP8, suggesting the involvement of ARP8 in 11q23 chromosomal abnormalities in ATM-deficient cells (Figure 5A and B). This finding was confirmed by the FISH analysis with a different DNA probe set (Figure 5—figure supplement 1A). In contrast, the number of split signal positive cells among the ATM-proficient 11–4 cells was increased by the depletion of ARP8 (Figure 5B). This was confirmed by the FISH analysis of ARP8-deficient Nalm-6 cells (Figure 5—figure supplement 1B). These findings suggest that ATM prevents the etoposide-induced 11q23 chromosome translocations through the regulation of ARP8.

**Figure 5—figure supplement 1.**
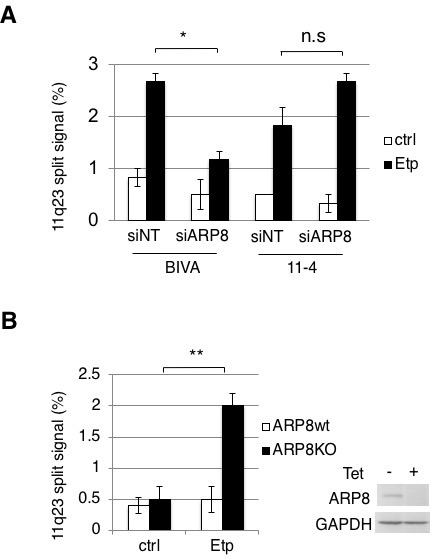
Dual-color FISH analysis of chromosome 11q23 using a different DNA probe set. (**A**) Dual-color FISH analysis of chromosome 11q23 in AT5BIVA and 11–4 cells, using the probe from Vysis. The non-targeting control siRNA (siNT) or siARP8-depleted cells were treated with etoposide for 15 min, washed, and cultured for 6 hr in fresh medium. Values represent the means ± SE from three independent experiments. *p<0.05. n.s: no significant difference. Source data are presented in Figure 5—source data 2. (**B**) Dual-color FISH analysis of chromosome 11q23 in wild-type or ARP8-knockout Nalm-6 cells. The cells were treated with 20 μM of etoposide for 15 min, and then cultured for 4 hr in fresh medium. In every experiment, 200 cells were analyzed. The average percentages of the cells with split signals are shown from four independent experiments. Values represent the means ± SE. **p<0.01, as determined by the Z test. The tetracycline-induced ARP8 knockout is shown in the gel image on the right. Source data are presented in Figure 5—source data 2.

Next, to investigate the role of the ATM-dependent phosphorylation of ARP8 in preventing the 11q23 chromosome abnormalities, we performed the FISH analysis of ATM-deficient BIVA cells expressing the phospho-mimicking ARP8 S412D mutant. The FISH analysis revealed that the expression of ARP8 S412D reduced the number of cells exhibiting split FISH signals after etoposide treatment in ATM-deficient cells (Figure 5C). In contrast, the expression of the phospho-deficient ARP8 S412A mutant increased the incidence of 11q23 chromosome translocations in ATM-proficient 11–4 cells (Figure 5D). Notably, ATMi treatment failed to enhance the event in ARP8 S412A mutant expressing cells (Figure 5E). Taken together, these findings strongly suggest that the ATM-dependent phosphorylation of ARP8 is required to prevent the etoposide-induced 11q23 chromosome abnormalities, through the negative regulation of RAD51 and INO80 binding to the BCR.

### ATM, but not ATR, negatively regulates RAD51 loading onto the BCR after etoposide treatment to repress 11q23 chromosome translocations

**Figure 6.**
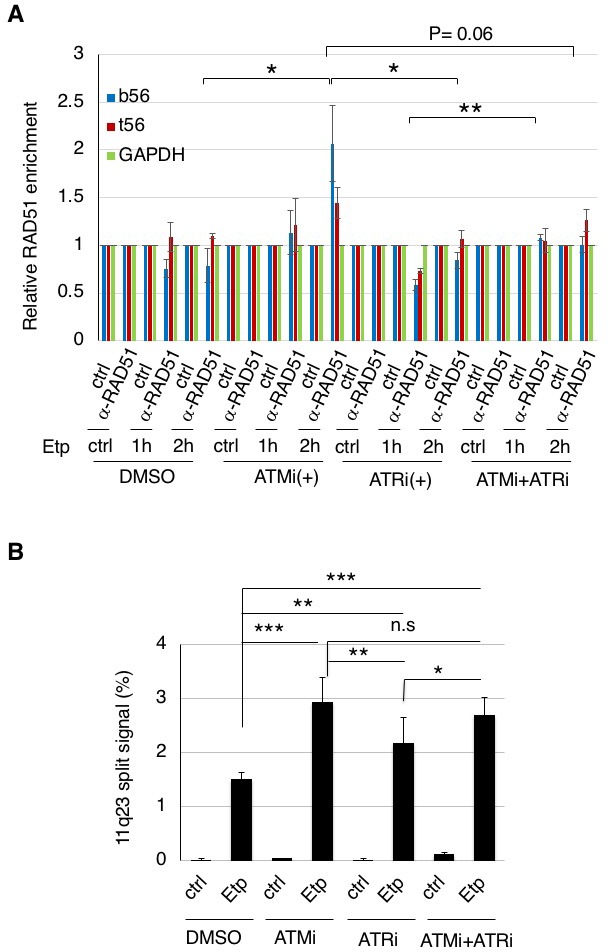
ATM, but not ATR, negatively regulates RAD51 loading onto the BCR after etoposide treatment to repress 11q23 chromosome translocations. (**A**) ChIP analysis of the RAD51 loading onto the MLL BCR in ATMi or ATRi or a combination of ATMi and ATRi treated 11–4 cells. 11–4 cells were treated with ATMi (10 µM), ATRi (10 µM), or a combination of ATMi and ATRi for 2 hr before etoposide treatment. After washing the cells, the inhibitors (5 μM) were added until the cells were harvested. The ChIP analysis was performed as described in Figure 4. Values represent the means ± SE from three independent experiments. *p<0.05. **p<0.01. Source data are presented in Figure 6—source data 1. (**B**) The percentages of 11–4 cells with split chromosome 11q23 signals are shown. 11–4 cells were treated with ATMi (10 µM), ATRi (10 µM), or a combination of ATMi and ATRi for 2 hr before etoposide treatment. After the cells were washed, the inhibitors (5 μM) were added until the cells were harvested. Dual-color FISH analyses of chromosome 11q23 were performed as described in Figure 5. Values represent the means ± SE from three independent experiments. *p<0.05. **p<0.01, ***p<0.001, n.s.: no significant difference. 10.7554/eLife.32222.026Figure 6—source data 1.Source raw data for Figure 6A and B.

Since the etoposide-induced phosphorylation of ARP8 was strongly repressed by an ATR inhibitor in 11–4 cells (Figure 1E), the ATR-dependent phosphorylation of ARP8 could also be involved in the excessive RAD51 loading onto the BCR. To test this possibility, we performed a chromatin immunoprecipitation assay of 11–4 cells treated with the ATR inhibitor, VE821. In contrast to the significant increase of etoposide-induced RAD51 binding to the BCR by the treatment with the ATM inhibitor, ATR inhibition failed to so (Figure 6A). These results suggest that ATR is not involved in the regulation of RAD51 binding at the BCR of MLL after etoposide treatment.

Next, we examined the effect of an ATRi on the etoposide-induced 11q23 chromosome translocations in ATM-proficient 11–4 cells, by the dual color FISH analysis using the MLL gene probes (Figure 6B). The increase of the split signal positive cells by ATRi was less than that by ATMi. Although ATR is suggested to be the major kinase responsible for ARP8 phosphorylation after etoposide treatment, this finding suggests that the effect of ATRi on the etoposide-induced chromosome translocations is limited. Moreover, no additional effects of ATRi on the increase of the chromosome translocations by ATMi were observed. Taken together, these findings strongly suggest that ATM, but not ATR, negatively regulates RAD51 loading onto the BCR after etoposide treatment to repress11q23 chromosome translocations.

## Discussion

Our present results are the first to identify ARP8 as the phosphorylation target regulated by ATM and ATR after the induction of DNA damage by etoposide treatment. ARP8 facilitates the binding of INO80 and RAD51 to the BCR of the MLL gene, while the phosphorylation of ARP8 suppresses it through a reduction of its interaction with INO80. The incidence of etoposide-induced 11q23 translocations is reduced by the expression of the phospho-mimicking ARP8 mutant in ATM-deficient cells. Moreover, the expression of the phospho-deficient ARP8 in ATM-proficient cells increases chromosome translocations. ATR was not involved in the regulation of RAD51 binding to the BCR after etoposide treatment. These findings strongly suggest that ATM represses the 11q23 chromosome translocations by regulating the binding of INO80 and RAD51 to the BCR of the MLL gene at functionally appropriate levels, via the phosphorylation of ARP8.

In response to DNA damage, the DNA repair process is facilitated by the phosphorylation of various proteins, including DNA repair factors, cell cycle regulators, and chromatin remodeling factors, through the positive regulation by ATM and ATR (Maréchal and Zou, 2013) (Cimprich and Cortez, 2008) (Clouaire et al., 2017; Shiloh, 2003; Shiloh and Ziv, 2013). In contrast, Exo1, an exonuclease for end resection in HR, is also phosphorylated by ATM in response to DNA damage, but to inhibit its exonuclease activity for the prevention of the untimely generation of ssDNA for RAD51 loading (Bolderson et al., 2010). In this study, we show that the phosphorylation of ARP8 represses the binding of INO80 and RAD51 to damaged chromatin (Figures 2 and 4). Importantly, the phosphorylation of ARP8 reduced its interaction with INO80 (Figure 3, Figure 3—figure supplements 1 and 2). Since ARP8 is required for the binding of the INO80 complex to damaged chromatin (Kashiwaba et al., 2010; Saravanan et al., 2012), the reduced interaction of ARP8 with INO80 by its phosphorylation may repress the binding of INO80 to the damaged chromatin and thus reduce ssDNA formation and RAD51 loading around damaged sites. ARP8 phosphorylation is significant from 2 hr after etoposide treatment, which is slower than the phosphorylation of ATM and H2AX. Therefore, it may also facilitate the dissociation of INO80 from damaged chromatin after the appropriate remodeling of damaged chromatin for DNA repair to avoid illegitimate recombination, leading to chromosome abnormalities. The phosphorylation of various proteins regulated by ATM may play an important role to prevent chromosome abnormalities, by maintaining the recombination activity within an appropriate range through both positive and negative regulation of repair proteins.

The phosphorylation of ARP8 is relatively slow, and occurs more than 2 hr after etoposide treatment, as compared to the early activation of ATM within 1 hr (Bakkenist and Kastan, 2003; Tanaka et al., 2007). Since the recombinational repair of DSBs starts slowly as compared to the end-joining repair, which normally begins within 30 min (Mao et al., 2008), this is consistent with the notion that ARP8 plays an important role in the appropriate regulation of the recombinational repair proteins. The slower phosphorylation of ARP8 also suggests the involvement of kinases other than ATM. Indeed, we found that ATR also regulates the etoposide-induced phosphorylation of ARP8. Moreover, ATRi repressed the phosphorylation of ARP8 especially from 2 hr after the etoposide treatment. These findings suggest the presence of different regulation systems of ARP8 phosphorylation after the induction of DNA damage. The phosphorylation of ARP8 after etoposide treatment could be regulated by multiple steps and factors for the precise control of DNA repair activity to maintain chromosome stability.

This study revealed that ATR is likely to be the major kinase responsible for the etoposide-induced ARP8 phosphorylation. However, unlike the inhibition of ATM, ATR inhibition failed to increase the RAD51 binding to the BCR of MLL after etoposide treatment (Figure 6), suggesting that ATR is not involved in the regulation of RAD51 binding to the BCR after etoposide treatment. Although the mechanism of 11q23 chromosome translocations is still unclear, a specific DNA sequence and/or chromatin structure of the BCR has been suggested to promote the mis-rearrangement of this region during the repair process (Gole and Wiesmüller, 2015). Therefore, ARP8 phosphorylation by ATM functions in the prevention of chromosome translocation at the BCR, while that by ATR may play roles in the repair of different types of DNA damage not relevant to chromosome translocations. Further studies are required to clarify the distinct roles of ATM and ATR in the phosphorylation of ARP8 after the induction of DNA damage, to coordinate the HR activity for accurate DNA repair.

Several lines of evidence have suggested the association of HR with genomic instability (Bishop and Schiestl, 2003; Guirouilh-Barbat et al., 2014; Mizuno et al., 2009; Reliene et al., 2007; Ruiz et al., 2009). RAD51-deficient vertebrate cells accumulate chromosomal breaks, resulting in an early embryonic lethal phenotype (Lim and Hasty, 1996; Sonoda et al., 1998). However, the overexpression of human RAD51 also leads to genomic instability (Kim et al., 2001; Marsden et al., 2016; Reliene et al., 2007; Richardson et al., 2004). These findings suggest that both the down- and up-regulation of the recombination activities through the levels of the RAD51 protein are associated with chromosomal instability. Together with the regulation of the RAD51 function at the protein level, the regulation of the BCR binding by the ATM-regulated phosphorylation of ARP8 may play an important role in maintaining the local recombination activities within an appropriate range, to ensure the fidelity of DNA repair and prevent chromosome translocations. INO80 and ARP8 have been shown to regulate the RAD51 loading to damaged chromatin in yeast (Tsukuda et al., 2005) (Tsukuda et al., 2009) (van Attikum et al., 2007) (Lademann et al., 2017). Moreover, the overexpression of human RAD51 leads to the various types of chromosome abnormalities (Reliene et al., 2007; Richardson et al., 2004). Therefore, this regulation of HR by ARP8 in human cells may also be applicable to DSBs in general.

The wild-type and the phospho-deficient mutant of ARP8 show increased interactions with INO80 after etoposide treatment, but the phospho-mimetic mutant does not (Figure 3, and Figure 3—figure supplements 1 and 2). Human ARP8 contains five insertions in the conserved actin fold domain (Gerhold et al., 2012) (Figure 1—figure supplement 1B). The S412 residue is located in a major loop insertion (insertion IV, residues 401–507) (Figure 1—figure supplement 1C). This region lacks interpretable electron density, suggesting its high flexibility to mediate dynamic protein–protein interactions (Gerhold et al., 2012). Moreover, the involvement of insertion IV in forming a proper ARP8-DNA complex has been suggested (Osakabe et al., 2014). Therefore, the phosphorylation of S412 within insertion IV of ARP8 could affect the binding activity of the INO80 complex to damaged chromatin, by suppressing the interactions with INO80 and components of damaged chromatin. Interestingly, ARP8 is conserved from yeast to human, but the 412-SQ motif is conserved only in higher eukaryotes, and not in yeast, Drosophila and Xenopus (Figure 1—figure supplement 1D). Although the mechanism by which the ARP8 phosphorylation regulates the activity of the INO80 complex remains to be clarified, the DNA damage-dependent ARP8 phosphorylation may have evolutionary advantages in DNA repair.

Chromosome abnormalities involving the MLL gene are one of the most frequent chromosomal aberrations observed in secondary leukemia associated with cancer therapy. We have shown that the etoposide-induced DNA damage in ATM-deficient cells facilitates the illegitimate recombination at the MLL gene, through the excessive binding of RAD51 and INO80 to the BCR. Our findings highlight the importance of the ATM-dependent modulation of recombination repair to avert 11q23 chromosome translocations. Further studies to investigate the mechanism that maintains the fidelity of DNA repair activity, using the chromosome translocations observed in secondary malignancy will provide new insights into the general mechanisms of carcinogenesis.

## Materials and methods

**Key resources table:** 

Reagent type (species) or resource	Designation	Source or reference	Identifiers	Additional information
Gene (*Homo Sapiens*)	arp8	NA	GenBank: GeneID 93973	
Gene (*Homo Sapiens*)	ino80	NA	GenBank: GeneID 54617	
Gene (*Homo Sapiens*)	atm	NA	GenBank: GeneID 472	
Gene (*Homo Sapiens*)	atr	NA	GenBank: GeneID 545	
Gene (*Homo Sapiens*)	rad51	NA	GenBank: GeneID 5888	
Gene (*Homo Sapiens*)	rpa2	NA	GenBank: GeneID 6118	
Cell line (*Homo Sapiens*)	AT5BIVA	PMID:21048951	RRID: CVCL_7442	Cell line maintained in S. Matsuura lab;
Cell line (*Homo Sapiens*)	11–4	PMID:21048951		Cell line maintained in S. Matsuura lab;
Cell line (*Homo Sapiens*)	U2OS	ATCC	RRID: CVCL_0042	
Cell line (*Homo Sapiens*)	GM0637	other	RRID: CVCL_7297	Cell line maintained in T. Cremer lab;
Cell line (*Homo Sapiens*)	Tet-Off ARP8 Nalm-6	PMID:25299602		Cell line maintained in M. Harata lab;
Transfected construct (*Homo Sapiens*)	Flp-In T-REx 11–4	this paper		Progenitor = 11–4; constructed by use of Flp-In T-REx core kit (Invitrogen)
Transfected construct (*Homo Sapiens*)	Flp-In T-REx 11–4 HA-ARP8-WT	this paper		Progenitor = Flp In T-REx 11–4; Addition of tetracyclin induces expression of HA-tagged recombinant ARP8 wild-type protein
Transfected construct (*Homo Sapiens*)	Flp-In T-REx 11–4 HA-ARP8-S412A	this paper		Progenitor = Flp In T-REx 11–4; Addition of tetracyclin induces expression of HA-tagged recombinant ARP8-S412A mutant protein
Transfected construct (*Homo Sapiens*)	U2OS HA-ARP8-WT	this paper		Progenitor = U2 OS; stably expressing HA-tagged recombinant ARP8 wild-type protein
Transfected construct (*Homo Sapiens*)	U2OS HA-ARP8-S412A	this paper		Progenitor = U2 OS; stably expressing HA-tagged recombinant ARP8-S412A protein
Antibody	anti-phospho ATM/ATR substrate motif (rabbit monoclonal)	Cell Signaling Technology	Cell Signaling Technology:Cat# 6966S; RRID:AB_10949894	
Antibody	anti-ATM protein kinase pS1981 (mouse monoclonal)	Rockland	Rockland:Cat# 200-301-400; RRID:AB_217868	
Antibody	anti-HA Tag (mouse monoclonal)	Merck Millipore	Merck Millipore:Cat# 05–904; RRID:AB_417380	
Antibody	anti-Histone H2A.X, phospho (Ser139) (mouse monoclonal)	Merck Millipore	Merck Millipore:Cat# 05–636; RRID:AB_309864	
Antibody	anti-human RAD51 (rabbit polyclonal)	Bio Academia	Bio Acdemia:Cat# 70–001; RRID:AB_2177110	
Antibody	anti-phospho RPA32 (S4/S8) (rabbit polyclonal)	Bethyl Laboratories	Bethyl Laboratories:Cat# A300-245A; RRID:AB_210547	
Antibody	anti-INO80 (rabbit polyclonal)	Bethyl Laboratories	Bethyl Laboratories:Cat# A303-371A; RRID:AB_10950580	
Antibody	anti-Human RPA/p34 (Replication Protein A) Ab-1 (mouse monoclonal)	Lab Vision	Lab Vision:Cat# MS-691-P0; RRID:AB_143149	
Antibody	anti-GAPDH (mouse monolconal)	Santa Cruz Biotechnology	Santa Cruz Biotechnology: sc-32233; RRID:AB_627679	
antibody	anti-Histone H2A.X	Abcam	Abcam: Cat# ab124781; RRID_AB_10971675	
antibody	anti-beta-actin (mouse monoclonal)	Sigma-Aldrich	Sigma-Aldrich:Cat # A5441; RRID:AB_476744	
antibody	anti-ARP8 (rabbit monoclonal)	PMID:25299602		
antibody	Cy3-secondary	Invitrogen		
Recombinant DNA reagent	pME18FL-hARP8	PMID:18163988		
Recombinant DNA reagent	pcDNA3.1/Myc-His(-)	Invitrogen	Invitrogen:Cat # V855-20	
Recombinant DNA reagent	pcDNA5/FRT/TO	Invitrogen	Invitrogen:Cat # K650001	
Recombinant DNA reagent	HA-arp8	this paper		Progenitor = pME18FL-hARP8; PCR, HA tag was fused; mutagenized in the Ambion Silencer Select s41201 siRNA target site for resistance; inserted into pcDNA3.1/Myc-His(-) or pcDNA5/FRT/TO
Recombinant DNA reagent	HA-arp8-S412A	this paper		Progenitor = HA-arp8; PCR, mutagenized; inserted into pcDNA3.1/Myc-His(-) or pcDNA5/FRT/TO
Recombinant DNA reagent	HA-arp8-S412D	this paper		Progenitor = HA-arp8; PCR, mutagenized; inserted into pcDNA3.1/Myc-His(-)
Sequence-based reagent	oligonucleotide for construction of siRNA-resistant HA-ARP8	this paper		5'-CTCAACAAAATGCCACCATCGTTCAGACGTATAATTGAAAATGTGGATG-3'
Sequence-based reagent	oligonucleotide for construction of siRNA-resistant HA-ARP8-S412A	this paper		5'-TTGCAGCACAGAGCTCAGGGCGATCCTG-3'
Sequence-based reagent	oligonucleotide for construction of siRNA-resistant HA-ARP8-S412D	this paper		5'-TTGCAGCACAGAGATCAGGGCGATCCTG-3'
Sequence-based reagent	primer for RT-PCR of bt56 forward	PMID:21048951		5'-TACTCTGAATCTCCCGCA-3'
Sequence-based reagent	primer for RT-PCR of bt56 reverse	PMID:21048951		5'-CGCTCGTTCTCCTCTAA-3'
Sequence-based reagent	primer for RT-PCR of t56 forward	PMID:21048951		5'-TTGCCAAGTCTGTTGTGAG-3'
Sequence-based reagent	primer for RT-PCR of t56 reverse	PMID:21048951		5'-CAGAGGCCCAGCTGTAGTTC-3'
Sequence-based reagent	primer for RT-PCR of GAPDH forward	this paper		5'-TCTCCCCACACACATGCACTT-3'
Sequence-based reagent	primer for RT-PCR of GAPDH reverse	this paper		5'-CCTAGTCCCAGGGCTTTGATT-3'
Sequence-based reagent	primer for RT-PCR of beta-globin forward	PMID:21048951		5'-TTGGACCCAGAGGTTCTTTG-3'
Sequence-based reagent	primer for RT-PCR of beta-globin reverse	PMID:21048951		5'-GAGCCAGGCCATCACTAAAG-3'
Commercial assay or kit	Duolink PLA	Sigma-Aldrich	Sigma-Aldrich:Cat # DUO92002, DUO92004, DUO92008	Proximity Ligation Assay
Chemical compound, drug	etoposide	Sigma-Aldrich	Sigma-Aldrich:Cat # E1383	
Chemical compound, drug	ATM inhibitor (KU55933)	Merck Millipore	Merck Millipore:Cat# 118500	
Chemical compound, drug	ATR inhibitor IV	Merck Millipore	Merck Millipore:Cat# 504972	
Software, algorithm	Metefer 4 MetaCyte	Metasystems	v 3.11.4	software for FISH analysis
Software, algorithm	Image J	NIH		
Other	XL MLL Plus	Metasystems Probes	Metasystems Probes:Cat# D5060-100-OG	probe for FISH analysis
Other	LSI MLL Dual Color, BreakApart Rearrangement Probe	Vysis, Abbott Molecular Inc.	Vysis, Abbott Molecular Inc.: 32–190083	probe for FISH analysis

### Cell culture and chemical treatment

The SV40-transformed AT fibroblast cell line AT5BIVA and its ATM-proficient derivative, AT5BIVA cells reconstituted with chromosome 11 (11-4), were kindly provided by Dr. S. Matsuura (Sun et al., 2010). The AT5BIVA cells were cultured in Dulbecco’s Modified Eagle’s medium (DMEM, Sigma-Aldrich), supplemented with 10% fetal bovine serum (FBS, Equitech-Bio, Kerrville, USA). The 11–4 cells were maintained in DMEM supplemented with 10% FBS and 0.2 mg/ml of G418 (Nacalai Tesque). The Flp-In T-Rex 11–4 cells were maintained in DMEM, supplemented with 10% tetracycline-free FBS (Sigma-Aldrich, St. Louis, USA), 10 μg/ml blasticidin S HCl (Gibco, Japan) and 40 μg/ml hygromycin B (Invitrogen, Carlsbad, USA). The human osteosarcoma U2OS cell line (ATCC) was cultured in Minimum Essential Medium Eagle (MEM, Sigma-Aldrich), supplemented with 10% FBS. GM0637 cells were cultured in DMEM, supplemented with 10% FBS. Tet-Off ARP8 Nalm-6 cells were cultured at 37°C in RPMI-1640, containing GlutaMAX-I (Gibco) and supplemented with 10% FBS. For the induction of the ARP8 knockout, tetracycline (Sigma-Aldrich) was added to the culture medium to a final concentration of 2 μg/ml (Osakabe et al., 2014). AT5BIVA, 11–4 cell lines were kindly provided by Dr. S. Matsuura laboratory, Hiroshima University, Japan. U2OS cell line was purchased from ATCC. GM0637 cell line was kindly provided by Dr. T. Cremer laboratory, LMU, Germany. Tet-Off ARP8 Nalm-6 cell line was kindly provided by Dr. M. Harata laboratory, Tohoku University, Japan. For the induction of DNA damage, as described elsewhere, the cells were exposed to 100 μM etoposide (Sigma-Aldrich), unless otherwise stated, for 15 min, washed, and cultured in fresh medium. Dimethylsulfoxide (DMSO) was used as the vehicle for etoposide, and was present in the cell cultures at a final concentration of 0.1%. Unless otherwise stated, both the ATM inhibitor KU55933 (Merck Millipore, Billerica, USA) and ATR inhibitor IV VE821 (Merck Millipore) were used at 10 μM for two hours before etoposide treatment and at 5 μM after the cells were washed, respectively.

### Antibodies

The antibodies used for chromatin immunoprecipitation, immunoblotting, and immunofluorescence staining were rabbit anti-phospho ATM/ATR substrate motif (Cell Signaling Technology, Danvers, USA), mouse anti-phospho ATM (Rockland, Pottstown, USA), mouse anti-HA, mouse anti-γH2AX (Merck Millipore), rabbit anti-RAD51 (Bio Academia,Japan), rabbit anti -RPA2 S4/8 and rabbit anti-INO80 (Bethyl, Montgomery, USA), mouse anti-RPA34 (Lab Vision, Fremont, USA), rabbit anti-glyceraldehyde-3-phosphate dehydrogenase (anti-GAPDH) (Santa Cruz Biotechnology, USA), rabbit anti-H2AX (Abcam, UK), and mouse anti-β-actin (Sigma-Aldrich) and rabbit anti-ARP8 (Osakabe et al., 2014).

### RNAi and plasmids

All siRNAs were Ambion Silencer Select siRNAs (Thermo Fisher Scientific,USA). The siRNAs were s41201 for ARP8, s57219 for ATM, s3638 for CK2, s29257 and s224310 for INO80. Select Negative Control siRNA was used as the control. The siRNA interference experiments were performed 2 days after transfection with 0.2–0.3 nM siRNA, using Lipofectamine RNAiMAX (Invitrogen). The pcDNA 3.1 vector bearing the HA-tagged ARP8 was constructed by inserting the PCR-amplified ARP8 cDNA into the *Not*I/*Hind*III sites, followed by inserting the PCR-amplified HA between the *Apa*I and *Xho*I sites of pcDNA3.1/Myc-His (-) C. Plasmid transfections were performed using the GeneJuice transfection reagent (Novagen, Billerica, USA), according to the manufacturer’s instructions. For rescue experiments, the siRNA-resistant ARP8 expression vector was co-transfected with the siRNA, using the Lipofectamine 2000 transfection reagent (Invitrogen) for 2 or 3 days. The mutant vectors were constructed by site-directed mutagenesis, using the indicated oligonucleotides: For the siRNA-resistant HA-ARP8 mutant, 5'-ctcaacaaaatgccaccatcgttcagacgtataattgaaaatgtggatg-3', for the HA-ARP8 S418A mutant, 5'-TTGCAGCACAGAGCTCAGGGCGATCCTG-3', and for the S418D mutant, 5'-TTGCAGCACAGAGATCAGGGCGATCCTG-3'. The sequences were confirmed using a BigDye Terminator v3.1 Cycle Sequencing Kit and an Applied Biosystems Genetic Analyzer, model 3130.

### Establishment of stably expressing cells

To generate cells stably expressing U2OS, the pcDNA 3.1 plasmid encoding the HA-tagged wild-type or S412A mutant of ARP8 was transfected using the GeneJuice transfection reagent, with G418 selection (Nacalai Tesque, Japan). After the confirmation of stable expression by immunofluorescence staining and immunoblotting, pools of single clones were used for experiments. For the generation of the inducible expression of wild-type or phosphorylation mutant ARP8 in 11–4 cells, the Flp-In System (Thermo Fisher Scientific) was used, according to the manufacturer’s instructions. The siRNA-resistant HA-tagged wild-type,or S412A ARP8 fragment from pcDNA 3.1 HA-ARP8 was inserted between the *Hind*III and *Kpn*I sites of the pcDNA5/FRT/TO vector. For the induction of the HA-ARP8 expression, a final concentration of 2 μg/ml tetracycline was added for 24 hr.

### FISH analysis

FISH analyses were performed using the 11q23 chromosome translocation probe (XL MLL plus, MetaSystems probes, Germany) and the LSI MLL Dual Color, BreakApart Rearrangement Probe (Vysis, Abbott Molecular Inc. Abbott park, USA), according to the manufacturers’ protocols. For the DNA FISH analysis using the probe from Metaystems, the images were acquired on an Axio Imager Z2 microscope (Carl Zeiss, Germany) equipped with the MetaSystems software. Subsequently, at least 2000 etoposide-exposed or DMSO-exposed cells were counted by the Metafer platform, MetaCyte, and the cells containing split signals (separated by >1 μm) were monitored. For the DNA FISH analysis using the probe from Vysis, the images were acquired on a Zeiss AxioplanII microscope using an AxioCamMRm controlled by Axiovision. At least 200 etoposide-exposed or DMSO-exposed cells were counted. All FISH analyses were repeated three times.

### Chromatin immunoprecipitation and real-time PCR assay

In brief, the cells were fixed by adding formaldehyde to a 1% final concentration for 10 min at 25°C. The cells were then sonicated to prepare chromatin suspensions of DNA fragments that were roughly 300–500 bps in length. Immunoprecipitations were performed using antibodies against INO80 and RAD51. Normal rabbit IgG was used as the negative control. Real-time PCR reactions were performed using SYBR premix Ex Taq (TAKARA, Japan). The dissociation curve analysis of the melting temperature of the amplified DNA showed that each primer set gave a single, specific product. The immunoprecipitation data were normalized to those of a control region in the GAPDH or β-globin gene, to correct for experimental variation. The relative immunoprecipitation value represents the ratio of the immunoprecipitated DNA after chemical treatment to the immunoprecipitated DNA after vehicle treatment. All ChIP analyses were repeated at least three times, and in each experiment, quantitative PCR reactions were performed in duplicate. Values represent the means ± SE. The primers for real-time PCR were: bt56 forward: 5 '-TACTCTGAATCTCCCGCA-3' bt56 reverse: 5'-CGCTCGTTCTCCTCTAA-3' t56 forward: 5'-TTGCCAAGTCTGTTGTGAGC-3' t56 reverse: 5'-CAGAGGCCCAGCTGTAGTTC-3'

GAPDH forward: 5'-TCTCCCCACACACATGCACTT-3'

GAPDH reverse: 5'-CCTAGTCCCAGGGCTTTGATT-3'.

β-globin forward: 5'-TTGGACCCAGAGGTTCTTTG3'

β-globin reverse: 5'-GAGCCAGGCCATCACTAAAG3'

### Immunofluorescence staining

After fixation in 4% paraformaldehyde in 1X phosphate-buffered saline (PBS) for 10 min at room temperature, the cells were permeabilized with 0.1% sodium dodecyl sulfate (SDS)−0.5% Triton X-100 in 1x PBS for 5 min. For the detection of HA-tagged ARP8, fixed cells were incubated for 30 min at 37°C with a mouse anti-HA antibody (1:600) in 1% bovine serum albumin (BSA)/1 XPBS. Cy3-conjugated goat anti-mouse (1:1,000, Invitrogen) antibodies were used as the secondary antibodies. Cells were mounted using Vectashield containing DAPI and observed with a BZ-X700 microscope (Keyence).

### Immunoprecipitation and immunoblotting

The nuclear fraction was prepared as described previously (Liu et al., 2015). The whole cells extracts were prepared using buffer C (20 mM HEPES pH 7.9, 400 mM NaCl, 1 mM EDTA, 0.1% NP-40, 0.5 mM DTT, 1X protease inhibitor (Roche), 1X phosphatase inhibitor (Nacalai Tesque), 20% glycerol), and the diluted lysates were used for immunoprecipitation. For immunoprecipitation assay of transfected cells, anti-HA antibody conjugated IgG Dynabeads (Novex) were used, and for the endogenous protein, anti-INO80 antibody, anti-ARP8 antibody, or normal IgG conjugated IgG Dynabeads were used. The immunoprecipitation analysis was performed at least twice to confirm the results. The precipitates were electrophoresed through a gel and probed by western blotting with the indicated the antibodies. The intensities of the bands were quantified, using the Image J software.

### Proximity ligation assay (PLA)

GM0637 cells were transfected with the HA-tagged wild type or S412A or S412D mutant of ARP8 or empty vector for 24 hr, fixed with 2% paraformaldehyde in PBS for 10 min at room temperature and permeabilized with 0.5% Triton X-100 in PBS for 5 min at room temperature. The cells were incubated with the rabbit anti-INO80 and mouse anti-HA antibodies diluted in 0.1% BSA for 30 min at 37°C in a moist chamber. Proximity ligation was then conducted in situ, according to the manufacturer’s instructions (Olink Bioscience, Sweden). We used the PLA probe anti-rabbit PLUS and the PLA probe anti-mouse MINUS. To visualize the interaction between two proteins, the samples were incubated for ligation and amplification. After serial SSC (sodium/sodium citrate) washes, nuclei were stained with DAPI. The slides were mounted with Vectashield (Vector Labs). PLA signals were detected with an LSM780 confocal microscope (Carl Zeiss), with a 63 × 1.40 NA plan-apochromat objective, and counted in at least 400 cells with the Image J software. All PLA analyses were repeated four times.

### Data analysis

Data in all experiments are represented as mean ± SE. Statistical analysis was performed using the two-tailed unpaired t- test. For the FISH analysis, the percentages of cells with split signals were determined by the Z test of homogeneity for independent samples. Results reaching p<0.05 were considered to be statistically significant (*p<0.05, **p<0.01, ***p<0.001).

## Additional files

Transparent reporting form

### Data availability

All data generated or analysed during this study are included in the manuscript and supporting files. Source data files have been provided.
